# Novel Remedy for Whooping-Cough

**Published:** 1874-08

**Authors:** J. E. Wendel


A Novel Remedy for Whooping-Cough.—*By Dr. J. E. Wendel.* "I was called three weeks ago to see a little white girl, seven years of age, who had had pertussis four weeks; had been coughing, whooping and vomiting furiously. I visited her to see what injuries she had received from falling from the second to the first floor of the dwelling, (fourteen feet) striking on her back on the floor. When I arrived she had recovered consciousness, but complained of some pain through the chest. The next day she was up, and has been to this time, and seems to be entirely cured; coughs almost none at all; does not whoop any. Three or four other children in the same family, who contracted the disease at the same time, are still coughing, whooping and vomiting. I do not recommend the remedy, but give it as one of the curiosities of my experience. The explanation of the cure is, I apprehend, easily given."—*American Practitioner.*

NOTE.—Last year we made a similar observation at Rome. A little girl five years old, who was suffering severely with whooping cough, fell, accidentally, down a flight of stairs, striking the head rather severely, but inflicting no external wound. The concussion of the brain, incident to the fall, eventuated some hours afterwards in convulsions, which were treated with chloral and potassic bromide. It was observed that the cough ceased entirely, and it was suspected that the chloral and bromide had *cured* it. The remedy was therefore given to several other children of the family, all of whom had the whooping-cough. The result clearly indicated that cerebral concussion is the remedy for whooping-cough, and not chloral nor bromide; thus adding another to the very numerous instances in which we have been taught to exercise extreme caution in reasoning from effect back to cause.—EDITOR.

